# Clinical Evidence on Resorbable Calcium Phosphate Biomaterials for Alveolar Bone Regeneration: A Scoping Review Focusing on Brushite, Monetite, and Tricalcium Phosphates

**DOI:** 10.3390/bioengineering13030366

**Published:** 2026-03-20

**Authors:** Francesco Bianchetti, Riccardo Fabozzi, Catherine Yumang, Paolo Pesce, Nicola De Angelis, Maria Menini

**Affiliations:** 1 Department of Surgical Sciences and Integrated Diagnostics, University of Genoa, 16121 Genova, Italy; 5410011@studenti.unige.it (F.B.); 5138519@studenti.unige.it (R.F.);; 2 Private Practice, 15011 Acqui Terme, Italy

**Keywords:** alveolar bone regeneration, brushite, monetite, calcium phosphates, dental implants

## Abstract

**Background:** While hydroxyapatite (HA) is considered stable and non-resorbable, other calcium phosphate phases such as Tricalcium Phosphate (TCP), Brushite, and Monetite are characterized by higher solubility and biodegradation rates. This review aims to map the clinical evidence of these resorbable phases. **Objective:** The aim of this scoping review was to map and synthesize the available clinical evidence on resorbable calcium phosphate phases, focusing on TCP-, brushite-, and monetite-based biomaterials in alveolar bone regeneration. The review evaluates clinical indications, surgical protocols, reported outcomes, and existing knowledge gaps. **Methods:** This scoping review was conducted in accordance with the PRISMA-ScR guidelines. A comprehensive literature search was performed in PubMed, MEDLINE, Scopus, and SCI Clarivate databases without language or time restrictions (from June 2025 to August 2025) using terms related to brushite, monetite, dicalcium phosphate anhydrous, ridge augmentation, bone regeneration, and dental implants. Clinical studies involving brushite- or monetite-based biomaterials used for alveolar bone regeneration were eligible, including randomized controlled trials, prospective cohort studies, and case series. Data were charted descriptively with respect to study design, patient characteristics, clinical scenario, biomaterials used, surgical approach, healing time, outcome measures, and reported complications. No meta-analysis or formal assessment of comparative clinical effectiveness was undertaken, in line with scoping review methodology. **Results:** Seven clinical studies were included. The identified evidence encompassed heterogeneous clinical scenarios, including post-extraction alveolar ridge preservation, localized ridge augmentation, and periodontal or intraosseous defects with relevance to future implant placement. Study designs, defect characteristics, biomaterial formulations, and outcome measures varied substantially. Across studies, brushite- and monetite-based materials were associated with new bone formation and progressive graft resorption, as assessed by clinical, radiographic, and histological outcomes. Direct comparisons between studies were not feasible due to methodological and clinical heterogeneity. **Conclusions:** The available literature on brushite- and monetite-based biomaterials in alveolar bone regeneration is limited and heterogeneous. Current evidence supports their biocompatibility and resorbable nature across different clinical contexts, but does not allow conclusions regarding comparative clinical effectiveness. This scoping review highlights important gaps in the literature, particularly the need for well-designed randomized clinical trials with standardized indications and outcome measures.

## 1. Introduction

Alveolar bone resorption following tooth extraction is a well-documented and clinically relevant phenomenon. During the first months after extraction, the alveolar ridge undergoes marked horizontal and vertical dimensional changes, with up to two-thirds of the total resorption occurring within the first three months and a reduction in ridge width that may reach 50% within one year [1].

More recent estimates report an average loss of approximately 3.8 mm in width and 1.2 mm in height within the first six months [2], findings that are consistent with those of Van der Weijden et al., who described a mean reduction of 3.9 mm in width and 1.7 mm in buccal height over the same period [3].

This remodeling process is largely explained by the unique anatomy and biology of the alveolar bone. The buccal plate is often thin and composed mainly of bundle bone, which is highly dependent on the presence of the tooth and periodontal ligament. After extraction, the loss of functional loading leads to rapid resorption and subsequent external remodeling, predominantly affecting the buccal aspect of the ridge [4].

The extent of resorption is further influenced by local anatomical and clinical factors, including initial buccal plate thickness, ridge morphology, bone density, and soft tissue characteristics [5,6].

From a clinical standpoint, post-extraction ridge resorption may compromise optimal implant positioning and esthetic outcomes. To mitigate these changes, a variety of alveolar ridge preservation and augmentation techniques have been proposed, most commonly involving the use of bone grafting materials and barrier membranes [7].

Alveolar ridge preservation (ARP) refers to procedures performed immediately after tooth extraction to minimize post-extraction bone resorption within the socket. In contrast, reconstructive ridge augmentation encompasses surgical techniques aimed at restoring horizontal and/or vertical bone volume in already resorbed ridges, typically through the use of bone grafts, barrier membranes, or guided bone regeneration (GBR) approaches prior to implant placement [8].

Systematic reviews and meta-analyses have shown that such interventions can reduce horizontal and vertical dimensional changes compared with spontaneous healing [9,10,11]. Nevertheless, the magnitude and clinical relevance of these effects remain variable, and in several analyses the reported differences fail to reach statistical significance [12,13,14,15].

However, to date, no systematic review has specifically focused on the clinical use of brushite and monetite for alveolar ridge augmentation, and existing reviews generally address calcium phosphate biomaterials as a broader and heterogeneous category.

Clinical outcomes following alveolar ridge preservation and augmentation are strongly influenced by multiple interacting factors, including defect morphology, surgical technique, timing of implant placement, and, critically, the choice of grafting biomaterial. Autogenous bone grafts are still considered the reference standard due to their osteogenic, osteoinductive, and osteoconductive properties; however, their use is limited by donor-site morbidity and a non-negligible complication rate [16,17].

Allografts and xenografts represent widely used alternatives, but their biological activity is mainly limited to osteoconduction, and concerns persist regarding biological safety, ethical considerations, and long-term remodeling behavior [18,19].

Among synthetic substitutes, calcium phosphate-based biomaterials such as hydroxyapatite (HA), beta-tricalcium phosphate (β-TCP), and alpha-tricalcium phosphate (α-TCP), which differ significantly in crystallographic structure, solubility product, and in vivo resorption kinetics. HA is characterized by very slow resorption and frequent persistence of residual particles, whereas β-TCP exhibits faster degradation but limited mechanical strength, restricting its indications primarily to non-load-bearing applications [20,21,22].

These characteristics have stimulated interest in more soluble calcium phosphate phases, particularly brushite (dicalcium phosphate dihydrate, DCPD) and monetite (dicalcium phosphate anhydrous, DCPA).

Brushite- and monetite-based biomaterials are characterized by higher solubility and faster resorption compared with HA, potentially enabling a closer synchronization between graft degradation and new bone formation [20,23].

However, relevant differences exist between the two phases. Brushite may partially convert into hydroxyapatite in vivo, thereby slowing its resorption over time, whereas monetite does not undergo this phase transformation and is generally degraded more rapidly [20,23,24].

While these properties may favor biological remodeling, they are also associated with potential limitations, including mechanical fragility, acidic degradation by-products, and the risk of premature resorption with possible loss of volume stability.

Encouraging clinical and histological outcomes have been reported for brushite- and monetite-based materials in post-extraction sockets and other localized defects [24,25].

However, the available clinical literature remains limited and heterogeneous, encompassing a wide range of study designs, biomaterial formulations, surgical protocols, and outcome measures. In addition, several studies investigate periodontal or intraosseous defects rather than alveolar ridge augmentation strictly defined, making it difficult to extrapolate findings to implant-related regenerative procedures.

It should also be acknowledged that terminology in this field is not always used consistently. Terms such as alveolar ridge augmentation, alveolar ridge preservation, and socket preservation are sometimes applied interchangeably, despite referring to related but distinct clinical concepts. Alveolar ridge augmentation generally describes surgical procedures aimed at increasing ridge volume in deficient sites, either horizontally, vertically, or both, often as a staged approach prior to implant placement. In contrast, alveolar ridge preservation or socket preservation refers to interventions performed immediately after tooth extraction with the aim of limiting post-extraction resorption and maintaining existing ridge dimensions.

In the present scoping review, the term alveolar ridge augmentation is used as an overarching concept encompassing both post-extraction ridge preservation/socket preservation procedures and localized augmentation techniques, provided that the clinical objective involves regeneration or maintenance of alveolar bone relevant to future implant placement. This approach was adopted to ensure conceptual consistency while capturing clinically comparable regenerative scenarios across the available literature.

To date, no review has specifically mapped and synthesized the clinical evidence on the use of brushite- and monetite-based biomaterials in alveolar bone regeneration across different indications and study designs. Existing reviews typically address calcium phosphate biomaterials as a broad and heterogeneous category, without distinguishing between specific crystalline phases or resorption behaviors. Given the emerging interest in highly resorbable calcium phosphates and the diversity of clinical applications reported, a scoping review approach is warranted.

Therefore, the aim of this scoping review is to systematically map the available clinical evidence on brushite- and monetite-based biomaterials used for alveolar bone regeneration, describing their clinical indications, surgical protocols, reported outcomes, and complications, and identifying gaps in the current literature to inform future research.

## 2. Materials and Methods

### 2.1. Review Design and Protocol

This review was conducted as a **scoping review** in accordance with the **PRISMA Extension for Scoping Reviews (PRISMA-ScR)** guidelines [26]. The scoping review methodology was selected to systematically map the breadth and characteristics of the available clinical evidence on brushite- and monetite-based biomaterials used for alveolar bone regeneration, in the presence of heterogeneous study designs, indications, and outcome measures.

### 2.2. Scoping Review Questions

In line with PRISMA-ScR recommendations, the review was guided by broad, exploratory research questions rather than a PICO-driven comparative framework. No PICO framework was applied, as the aim was not to assess intervention effectiveness but to map existing evidence. Specifically, the following questions were addressed:

1. **In which clinical scenarios** have brushite- and monetite-based biomaterials been used for alveolar bone regeneration?
2. **What surgical protocols** (e.g., defect type, use of membranes, healing time, timing of implant placement) have been reported?
3. **Which outcome measures** (clinical, radiographic, histological, patient-reported) have been used to evaluate these materials?
4. **What patterns of biomaterial resorption and bone formation** have been described?
5. **Which gaps and limitations** can be identified in the current clinical literature?

No attempt was made to compare clinical effectiveness or to establish causal relationships between interventions and outcomes.

### 2.3. Eligibility Criteria

Eligibility criteria were defined iteratively, consistent with scoping review methodology, to capture the full extent of relevant clinical evidence. Because this is a scoping review, patient-related eligibility criteria were not defined a priori by the authors but were derived from the protocols of the included clinical studies. When reported, studies generally excluded patients presenting with active oral infections, untreated periodontal disease, or other oral conditions that could interfere with normal bone healing and regeneration.

**Inclusion criteria**

Studies were considered eligible if they met the following criteria:

- **Population:** Adult human subjects undergoing procedures involving alveolar bone regeneration relevant to current or future dental implant placement.
- **Concept:** Studies utilizing resorbable calcium phosphate phases, including dicalcium phosphate dihydrate (Brushite), dicalcium phosphate anhydrous (Monetite), and tricalcium phosphate (TCP) or their biphasic combinations, were included. Pure sintered Hydroxyapatite (HA) studies were excluded due to their non-resorbable nature.
- **Context:** Clinical scenarios including post-extraction alveolar ridge preservation (socket preservation), localized alveolar ridge augmentation, or periodontal and intraosseous defects when the surgical objective involved alveolar bone regeneration relevant to implant therapy.
- **Study design:** Randomized controlled trials, prospective cohort studies, and case series were eligible. This inclusive approach was adopted to reflect the limited availability of randomized evidence and to map the existing clinical experience with these biomaterials.
- **Follow-up:** Minimum follow-up of three months.
- **Language:** Studies published in English or accompanied by an English translation suitable for accurate data extraction.

Although the primary research question focused on alveolar ridge augmentation procedures, studies involving periodontal, intraosseous, and furcation defects were also considered eligible when the surgical objective included localized alveolar bone regeneration relevant to ridge preservation or future implant placement. This broader inclusion strategy was adopted to capture clinically meaningful evidence on brushite- and monetite-based biomaterials in anatomically and biologically comparable regenerative contexts.

**Exclusion criteria**

Studies were excluded if they met any of the following criteria:

- In vitro studies, animal studies, or preclinical investigations.
- Clinical studies not involving calcium phosphate-based biomaterials.
- Studies evaluating calcium phosphate materials without a brushite or monetite phase relevant to the review objectives.
- Follow-up shorter than three months.
- Narrative reviews, systematic reviews, conference abstracts without sufficient data, or reports with unavailable full text.

### 2.4. Information Sources and Search Strategy

The electronic literature search was conducted between June 2025 and August 2025. These dates refer exclusively to the period during which the search was performed. No restrictions were applied regarding the publication year of the included studies. The final search was completed in August 2025. The following electronic databases were searched:

- PubMed
- MEDLINE
- Scopus
- SCI Clarivate

The search strategy combined keywords and Boolean operators related to brushite, monetite, and dicalcium phosphate phases with clinical terms relevant to alveolar bone regeneration, including “Alveolar Ridge Augmentation”, “Alveolar Bone Grafting”, “Bone Regeneration,” “Jawbone Augmentation,” “Dental Implants,” “Implant Success,” “Osseointegration,” “Randomized Controlled Trial,” “Clinical Study,” and “Comparative Study.”. No temporal restrictions were applied. With regard to language, studies published in English or accompanied by an English translation suitable for accurate data extraction were considered eligible.

### 2.5. Study Selection

The study selection process followed a two-stage screening approach. After removal of duplicates, titles and abstracts were screened independently by two reviewers (F.B. and R.F.) against the predefined eligibility criteria. Potentially relevant articles were retrieved in full text and assessed independently for inclusion.

Disagreements at any stage were resolved through discussion, and when consensus could not be reached, a third reviewer (N.D.A.) was consulted. Reasons for exclusion at the full-text stage followed the PRISMA-ScR guidelines. 

### 2.6. Data Charting Process

Data were charted independently and in duplicate using a predefined data extraction form, consistent with PRISMA-ScR guidance. The following variables were extracted:

- **Study characteristics:** author, year of publication, country, study design.
- **Participant characteristics:** number of patients, age, sex (when reported).
- **Clinical scenario:** socket preservation, staged ridge augmentation, simultaneous augmentation with implant placement, periodontal or intraosseous defects.
- **Biomaterial characteristics:** brushite- or monetite-based formulation, composite components, granule or cement form.
- **Surgical protocol:** flap design, membrane use, healing time, timing of implant placement.
- **Outcome measures:** clinical, radiographic, histological/histomorphometric, and patient-reported outcomes, when available.
- **Follow-up duration and reported complications.**

No hierarchy of outcomes (primary vs. secondary) was imposed, as the objective of this scoping review was descriptive mapping rather than assessment of clinical effectiveness.

In accordance with the exploratory nature of scoping reviews, outcome measures were charted as reported by the authors, without prior prioritization or weighting. During data charting and synthesis, studies were further categorized according to the **type of clinical evidence provided**, distinguishing between:

- **Direct clinical evidence**, derived from studies in which brushite- or monetite-based biomaterials were used as the primary grafting material;
- **Indirect clinical evidence**, derived from studies evaluating structurally or chemically related calcium phosphate biomaterials with relevance to brushite or monetite behavior.

For clarity and interpretative transparency, studies were categorized into: direct clinical evidence, defined as investigations in which brushite (dicalcium phosphate dihydrate) or monetite (dicalcium phosphate anhydrous) represented the primary grafting phase; and indirect clinical evidence, defined as investigations evaluating tricalcium phosphate (TCP), alpha- or beta-TCP, or biphasic calcium phosphate (HA/TCP) systems whose biological behavior may partially overlap with, but does not directly represent, pure brushite or monetite phases. This distinction was maintained throughout data synthesis to prevent inappropriate extrapolation across chemically and biologically distinct calcium phosphate systems.

Particular attention was paid to the crystalline phase of tricalcium phosphate (TCP) reported in each study. When specified in the original publication, the TCP phase was recorded as alpha-tricalcium phosphate (α-TCP) or beta-tricalcium phosphate (β-TCP). In studies evaluating biphasic calcium phosphate (BCP), the hydroxyapatite (HA)/TCP ratio and the specific TCP phase were extracted when available. If the original article did not clearly report the TCP crystalline phase or HA/TCP ratio, this was explicitly noted in the data extraction and synthesis to avoid overinterpretation.

### 2.7. Data Synthesis

Given the heterogeneity of study designs, clinical indications, biomaterial formulations, and outcome measures, data were synthesized **descriptively**. Studies were grouped according to **clinical scenario**, including:

- post-extraction alveolar ridge preservation (socket preservation),
- staged horizontal and/or vertical ridge augmentation,
- simultaneous augmentation at the time of implant placement.

Findings were summarized narratively, focusing on patterns of use, reported outcomes, and observed variability across studies. No quantitative synthesis or meta-analysis was attempted.

Given the limited number of clinical studies investigating pure brushite or monetite phases, studies evaluating composite or biphasic calcium phosphate biomaterials were included only when a brushite- or monetite-related phase was explicitly reported or when the material’s resorption behavior was directly relevant to the scope of this review. This distinction was maintained throughout data charting and synthesis to avoid inappropriate generalization.

### 2.8. Critical Appraisal of Evidence

In accordance with PRISMA-ScR recommendations, scoping reviews are not designed to conduct formal risk-of-bias assessments to evaluate clinical effectiveness or to exclude or rank evidence based on methodological quality. The aim of this scoping review was to map the available clinical literature on brushite- and monetite-related biomaterials and to describe the characteristics of that evidence. Therefore, no formal risk-of-bias tool (such as RoB 2.0, Newcastle–Ottawa Scale, or JBI checklists) was used for the purpose of evaluating comparative effectiveness or weighting findings. Instead, key methodological features of included studies—such as study design, sample size, follow-up duration, clinical indications, and outcome measures—were extracted and descriptively summarized to provide context, highlight heterogeneity, and identify gaps in the current literature.

## 3. Results

### 3.1. Study Selection and Characteristics of the Evidence Base

A total of 1815 records were identified through the electronic literature search. After removal of duplicates and screening of titles and abstracts, full-text assessment was performed for potentially eligible articles. Following application of the predefined inclusion criteria, seven clinical studies were included in this scoping review. The main reasons for exclusion at the full-text stage are summarized in Table 1. The key characteristics, objectives, and reported outcomes of the included studies are summarized in Table 2.

The included studies were published between 1985 and 2020 and comprised three randomized controlled clinical trials, three controlled or comparative clinical studies, and one small case series. Sample sizes ranged from two patients to multicenter randomized trials, with follow-up periods varying from six weeks to three years.

Considerable heterogeneity was observed across studies with respect to:

- clinical indications and defect morphology,
- anatomical sites treated,
- surgical protocols and use of adjunctive measures (e.g., membranes),
- outcome measures and follow-up duration.

Given this variability, findings are presented descriptively and grouped according to the clinical scenario in which brushite- or monetite-related biomaterials were applied.

### 3.2. Alveolar Ridge Preservation and Regeneration in Post-Extraction and Periodontal Defects

In accordance with the predefined categorization, included studies are presented by distinguishing between **Direct Evidence** (brushite- or monetite-based biomaterials) and **Indirect Evidence** (TCP-based or biphasic calcium phosphate materials). This distinction is maintained to preserve interpretative accuracy, given the significant differences in crystalline structure, solubility, and resorption kinetics among these calcium phosphate phases. Four studies investigated the use of calcium phosphate-based biomaterials in periodontal or intra-bony defects, including furcation defects and intrabony periodontal lesions.

Pepelassi et al. [24] evaluated a composite graft containing tricalcium phosphate (TCP; crystalline phase not specified in the original publication), plaster of Paris, and doxycycline in Class II and III furcation defects, reporting clinical and radiographic outcomes after six months.

Nery et al. [25] assessed biphasic calcium phosphate (BCP) ceramic (reported as HA/TCP composite; specific HA/TCP ratio and TCP crystalline phase not specified in the original publication) in comparison with autogenous bone grafting and open flap curettage in periodontal osseous defects, with outcomes reported over a three-year follow-up.

Deshoju et al. [26] investigated a zinc-substituted monetite-based scaffold (Sil-Oss^®^) in periodontal intrabony defects, reporting clinical, radiographic, and histological observations related to bone fill and tissue mineralization.

Baldock et al. [30] described the clinical application of tricalcium phosphate (TCP; crystalline phase not specified in the original publication) implants in periodontal osseous defects in two patients, reporting short-term clinical tolerance and limited regenerative outcomes. Across these studies, reported outcome measures included probing depth changes, clinical attachment levels, defect fill, and, in selected cases, histological assessment of mineralized tissue formation. Surgical protocols and defect characteristics varied substantially among investigations.

Three studies explored the application of monetite- or calcium phosphate-based biomaterials for alveolar ridge preservation following tooth extraction. Flores Fraile et al. [27] conducted a randomized comparative clinical trial evaluating a synthetic bone substitute composed of monetite, silica gel, PS-wollastonite, and calcium-deficient hydroxyapatite in comparison with bovine-derived xenograft for post-extraction ridge preservation in the maxilla. Clinical, radiographic, and histological outcomes were reported.

Kesmas et al. [28] presented a preliminary clinical study assessing biphasic calcium phosphate (BCP) combined with a resorbable collagen membrane (HA/TCP ratio and TCP crystalline phase not clearly specified in the original publication) for ridge preservation after extraction of maxillary central incisors, with outcomes reported up to 12 months. Tamimi et al. [29] compared monetite granules with bovine hydroxyapatite in post-extraction alveolar sockets, reporting histological and histomorphometric findings at six months.

Outcome measures in these studies included changes in ridge dimensions, histological assessment of new bone formation, residual graft material, and tissue mineralization. Healing times, membrane use, and anatomical locations differed across investigations.

Across all included studies, outcomes were reported using a combination of:

- clinical parameters (probing depth, clinical attachment levels, defect fill),
- radiographic assessments (ridge dimensions, defect resolution),
- histological or histomorphometric analyses (new bone formation, residual biomaterial, connective tissue),
- with no studies reporting patient-reported outcome measures.

Outcome definitions, measurement methods, and reporting time points varied markedly, limiting direct comparison between studies. Follow-up durations ranged from short-term pilot observations to long-term clinical assessments extending up to three years.

In line with the objectives of this scoping review and the heterogeneity of the available evidence, no quantitative synthesis or meta-analysis was undertaken. Instead, a descriptive synthesis was performed to map the existing clinical applications of brushite- and monetite-related biomaterials, the types of defects treated, the surgical approaches employed, and the outcomes reported in the literature.

### 3.3. Complications and Adverse Events

The reporting of complications and adverse events across the included studies was heterogeneous and inconsistent. Available evidence was derived from studies with different clinical indications, biomaterials, and follow-up durations; therefore, findings are presented descriptively.

Pepelassi et al. [24] reported uneventful healing and absence of major postoperative complications following the use of tricalcium phosphate in periodontal defects. Histological evaluation revealed persistence of residual graft particles embedded in connective tissue.

Nery et al. [25] similarly reported good clinical tolerance of biphasic calcium phosphate ceramics, with no inflammatory or systemic adverse events. Residual graft particles were consistently observed at follow-up.

Deshoju et al. [26] reported uneventful healing and absence of graft-related complications in sites treated with zinc-substituted monetite and brushite-related materials. Gradual resorption of monetite was described.

Flores Fraile et al. [27] evaluated monetite-based substitutes in post-extraction sockets and reported clinical safety outcomes comparable to other grafting materials, with no increase in postoperative complications. Histological findings indicated substantial replacement of graft particles by newly formed bone.

Kesmas et al. [28] reported stable healing and absence of inflammatory complications following ridge preservation with biphasic calcium phosphate and collagen membrane, although residual graft particles were frequently observed at re-entry.

Tamimi et al. [29] reported no intraoperative or postoperative complications associated with monetite granules in post-extraction sockets. All sites healed successfully, allowing subsequent implant placement.

Baldock et al. [30] reported good tolerance of tricalcium phosphate particles, with no inflammatory reactions; however, histological analysis demonstrated persistence of graft particles within connective tissue up to nine months.

Overall, the included studies reported a low incidence of clinical complications associated with calcium phosphate-based grafts. For brushite- and monetite-based materials, available evidence suggests satisfactory short- to mid-term clinical safety. However, conclusions are limited by heterogeneity among studies, small sample sizes, and the lack of direct comparative trials.

## 4. Discussion

### 4.1. Scope and Purpose of the Review

The present study was conducted as a scoping review, with the explicit aim of mapping and synthesizing the available clinical evidence on the use of brushite- and monetite-based biomaterials in alveolar bone regeneration. Unlike a systematic review focused on clinical effectiveness or comparative efficacy, this work did not seek to determine whether these materials perform better or worse than alternative grafting options or spontaneous healing. Instead, the objective was to describe how these biomaterials have been investigated in different clinical contexts, which material formulations have been used, which outcomes have been reported, and where relevant gaps in the literature remain.

Within this methodological framework, heterogeneity is an expected and informative feature of the evidence base. Variability in study design, clinical indication, biomaterial composition, surgical protocols, and outcome assessment reflects the exploratory and developmental nature of research on brushite and monetite, rather than a limitation to be corrected through data aggregation or exclusion. Accordingly, findings are interpreted descriptively and contextually, in line with the PRISMA Extension for Scoping Reviews.

### 4.2. Evidence Distribution Across Clinical Indications

An important aspect emerging from the mapped evidence is the distinction between different biological and surgical scenarios in which brushite- and monetite-based biomaterials have been applied. Although this scoping review adopted an overarching framework of “alveolar bone regeneration,” the included studies fall into biologically distinct categories that should not be interpreted interchangeably.

Post-extraction alveolar ridge preservation represents a contained healing environment in which the socket walls are partially or completely preserved. In this context, the graft material is primarily intended to modulate natural post-extraction remodeling, limit dimensional contraction, and support early bone formation prior to implant placement. Studies by Tamimi et al. [29] and Flores Fraile et al. [27] fall within this category. Reported outcomes predominantly concern histomorphometric bone formation, residual graft content, and maintenance of ridge dimensions.

In contrast, reconstructive ridge augmentation in healed sites aims to achieve horizontal and/or vertical bone gain in partially or severely deficient ridges. This scenario typically involves non-contained defects, reduced vascular supply, greater mechanical demands, and the need for space maintenance. Evidence specifically addressing this indication remains limited within the mapped literature, and extrapolation from socket preservation data is not biologically justified.

A third group includes periodontal and intrabony defects, as investigated by Pepelassi et al. [24], Nery et al. [25], and Deshoju et al. [26]. These defects differ further in pathophysiology, being associated with chronic inflammatory periodontal disease rather than post-extraction remodeling or ridge deficiency. Clinical endpoints in these studies primarily relate to probing depth reduction and attachment gain rather than ridge volume maintenance or implant site development.

Given these fundamental biological and therapeutic differences, outcomes reported across these indications cannot be directly compared. The present review therefore emphasizes descriptive mapping within each clinical scenario rather than cross-indication synthesis.

### 4.3. Characteristics of Investigated Biomaterials

A defining characteristic of the mapped literature is the substantial heterogeneity of biomaterial formulations investigated. Included studies evaluated brushite-based cements, monetite-based granules, zinc-substituted monetite composites, and composite or biphasic calcium phosphate systems containing brushite- or monetite-related phases. For example, Deshoju et al. investigated zinc-substituted monetite (Sil-Oss^®^) in intrabony periodontal defects [26], while Flores Fraile et al. examined composite monetite-based substitutes in post-extraction sockets [27]. Tamimi et al. specifically evaluated monetite granules (anhydrous dicalcium phosphate) in human extraction sites [29].

Not all materials can be considered chemically pure brushite or monetite, and this variability reflects the historical and translational evolution of calcium phosphate biomaterials rather than inconsistency in study selection. Differences in composition, resorption kinetics, and mechanical behavior are summarized in Table 2 which provides a framework for interpreting the biological behavior reported across studies. As illustrated in Table 2, brushite and monetite differ in chemical phase, resorption dynamics, volume stability, and handling characteristics, factors that may influence their selection for specific clinical applications.

While this material heterogeneity limits direct comparison between studies, it also offers insight into current research trajectories, including attempts to balance resorption rate, space maintenance, and biological integration. From a scoping perspective, documenting this diversity is essential to understanding the landscape of available evidence.

An important limitation identified during data charting was the inconsistent reporting of TCP crystalline phase in older clinical studies. Several investigations referred generically to “tricalcium phosphate” without specifying whether α-TCP or β-TCP was used, despite their known differences in solubility, resorption kinetics, and in vivo behavior. Similarly, in studies evaluating biphasic calcium phosphate (BCP), the HA/TCP ratio and the specific TCP phase were not always clearly reported. This lack of physicochemical detail limits precise interpretation of biological outcomes and highlights the need for standardized reporting of calcium phosphate composition in future clinical trials.

A critical interpretative consideration concerns the inclusion of biphasic calcium phosphate (BCP) materials containing hydroxyapatite (HA) in several of the mapped studies. HA is characterized by low solubility and prolonged in vivo persistence, and its presence significantly alters the overall degradation kinetics and remodeling profile of HA/TCP composites compared with highly resorbable pure phases such as brushite (dicalcium phosphate dihydrate) and monetite (dicalcium phosphate anhydrous).

In biphasic systems, the HA fraction contributes to structural stability and space maintenance but reduces the overall resorption rate relative to pure brushite or monetite formulations. Consequently, biological outcomes observed with HA-containing composites cannot be assumed to reflect the behavior of fully resorbable calcium phosphate phases.

This distinction is particularly relevant when interpreting histomorphometric outcomes such as residual graft percentage and new bone formation, as prolonged persistence of HA particles may influence both radiographic appearance and tissue remodeling patterns. Therefore, findings from HA/TCP composites should be considered indirect evidence with limited generalizability to pure brushite or pure monetite biomaterials.

### 4.4. Outcome Domains Reported in the Literature

Across the included studies, a broad range of outcome domains was reported, with considerable variation in assessment methods and follow-up duration. Histological and histomorphometric outcomes, such as the proportion of newly formed bone and residual graft material, were frequently described, particularly in studies by Tamimi et al. [29] and Flores Fraile et al. [27]. Radiographic outcomes, including changes in ridge dimensions and radiodensity, were also commonly reported, as seen in the work of Kesmas et al. [28].

Clinical parameters, such as probing depth reduction, clinical attachment level gain, and feasibility of subsequent implant placement, were reported primarily in periodontal and intrabony defect studies, including those by Pepelassi et al. [24], Nery et al. [25], and Deshoju et al. [26]. In contrast, patient-reported outcome measures, esthetic evaluations, and long-term functional outcomes were rarely included.

Adverse events and healing patterns were generally reported descriptively, with most studies noting uneventful healing and a low incidence of complications, including those by Tamimi et al. [29] and Flores Fraile et al. [27]. The lack of standardized outcome definitions and measurement protocols across studies represents a key feature of the existing literature. Within the scoping review framework, outcomes were therefore mapped descriptively, without prioritization or hierarchical ranking.

### 4.5. Methodological Considerations and Limitations of the Evidence

The body of evidence mapped in this scoping review is limited in size and characterized by substantial heterogeneity. Only seven clinical studies met the inclusion criteria, encompassing randomized controlled trials, prospective clinical studies, and small case series. Sample sizes ranged from pilot investigations involving a limited number of participants to larger multicenter trials, and follow-up duration varied considerably across studies.

Variability was observed in clinical indications, defect morphology, biomaterial composition, surgical protocols, membrane use, healing periods, and outcome assessment methods. In addition, reporting of patient characteristics, defect classification, and surgical details was not uniform across investigations, further limiting cross-study comparability.

In accordance with PRISMA-ScR guidance, this review did not aim to evaluate comparative effectiveness nor to perform a formal risk-of-bias assessment. The objective was descriptive mapping of the extent, nature, and characteristics of the available clinical literature on brushite- and monetite-related biomaterials. Consequently, findings should be interpreted within the exploratory scope of the review and not as evidence of established clinical superiority or definitive therapeutic recommendations.

The limited number of studies, the diversity of investigated biomaterial formulations, and the absence of standardized outcome reporting highlight the early developmental stage of clinical research in this field.

Throughout this review, a clear distinction has been maintained between direct clinical evidence derived from pure brushite or monetite phases and indirect evidence derived from TCP-based or HA/TCP composite systems. While indirect evidence may provide contextual insights into calcium phosphate behavior, it cannot be considered equivalent to phase-specific validation of highly resorbable brushite or monetite biomaterials. Interpretations and clinical inferences should therefore be limited to the level of evidence provided within each category.

### 4.6. Implications for Future Research

Future research should adopt indication-specific study designs. Socket preservation trials should focus on dimensional stability and implant-related outcomes, whereas reconstructive ridge augmentation studies should specifically evaluate horizontal and vertical bone gain, volume stability, and long-term implant success in non-contained defects. Conflation of these indications risks obscuring clinically meaningful differences in biomaterial performance. The evidence mapping performed in this scoping review highlights several knowledge gaps. There is a limited number of indication-specific randomized controlled trials, particularly for staged augmentation procedures and implant-associated applications. Standardized outcome sets, including clinically meaningful endpoints, patient-reported outcomes, and esthetic parameters, are largely absent. Long-term clinical data extending beyond early healing phases remain scarce.

An additional and clinically relevant gap identified in the mapped literature is the complete absence of Patient-Reported Outcome Measures (PROMs). None of the included studies incorporated validated instruments to assess patient-centered outcomes such as postoperative pain intensity, functional impairment, oral health-related quality of life, esthetic perception, or overall treatment satisfaction.

Given that alveolar ridge preservation and reconstructive augmentation procedures are often elective and closely linked to implant rehabilitation and esthetic expectations, evaluation of patient experience should be considered an essential component of clinical research. The exclusive reliance on surrogate biological endpoints—such as percentage of newly formed bone or residual graft material—does not fully capture treatment impact from the patient’s perspective.

Future clinical trials investigating brushite- and monetite-based biomaterials should therefore integrate validated PROM instruments alongside radiographic and histomorphometric outcomes. The inclusion of standardized patient-centered endpoints would enhance clinical interpretability, improve comparability across studies, and align research design with contemporary principles of evidence-based and value-based healthcare.

Future investigations would benefit from well-designed, adequately powered clinical studies with clearly defined indications, detailed reporting of biomaterial composition and surgical protocols, and harmonized outcome measures. Such efforts would facilitate more focused evidence synthesis and enable future systematic reviews to address questions of comparative effectiveness.

### 4.7. Paucity of High-Level Evidence for Pure Brushite and Monetite Phases

A critical finding emerging from this scoping review is the marked scarcity of high-level clinical evidence specifically investigating chemically pure brushite or pure monetite phases in alveolar bone regeneration. Of the seven included studies, only a limited subset evaluated materials that can be considered predominantly monetite-based, while several investigations assessed composite formulations or biphasic calcium phosphate systems in which the exact crystalline composition was either mixed or incompletely specified.

Furthermore, the available studies are few in number and largely characterized by small sample sizes, heterogeneous clinical indications, and limited long-term follow-up. Randomized controlled trials specifically designed to isolate the biological and clinical performance of pure brushite or monetite in clearly defined augmentation scenarios are currently lacking.

As a consequence, the present review should not be interpreted as evidence of established clinical effectiveness for these phases, but rather as a mapping of early-stage clinical exploration. The limited volume and methodological variability of the available literature underscore the need for rigorously designed, adequately powered, indication-specific randomized clinical trials before definitive conclusions can be drawn.

## 5. Conclusions

This scoping review provides a structured synthesis of the current clinical evidence on brushite- and monetite-based biomaterials for alveolar bone regeneration. The available literature shows that these materials have been mainly investigated in alveolar ridge preservation and in periodontal or intrabony defects, while evidence for staged ridge augmentation and especially for simultaneous implant-associated procedures remains scarce. Across indications, studies display marked heterogeneity in design, defect characteristics, biomaterial formulations, surgical protocols, and outcome assessment, reflecting the exploratory stage of research in this field.

Reported outcomes include histological, histomorphometric, radiographic, and clinical parameters, with a predominance of surrogate endpoints such as percentage of newly formed bone and residual graft material. Adverse events were infrequently described and healing was generally reported as uneventful; however, standardized definitions of complications and long-term implant-related outcomes were largely absent. Patient-reported outcomes and esthetic parameters were rarely assessed.

Several limitations must be clearly acknowledged. First, as a scoping review, this work does not aim to evaluate comparative effectiveness, and no quantitative synthesis or meta-analysis was performed. Second, the included studies are few in number and often characterized by small sample sizes, short follow-up, and predominantly non-randomized designs. Third, heterogeneity in biomaterial composition and outcome reporting limits cross-study comparability. Finally, incomplete reporting of surgical and patient-related variables in primary studies restricts external validity.

This scoping review mapped both direct and indirect clinical evidence related to brushite- and monetite-based biomaterials. However, direct high-level evidence specifically investigating pure brushite or monetite phases remains limited, and a substantial portion of the literature derives from TCP-based or biphasic systems that differ significantly in physicochemical behavior.

Therefore, no definitive conclusions can be drawn regarding the superiority or clinical effectiveness of brushite- or monetite-based materials. Instead, this review highlights the need for well-designed, indication-specific randomized controlled trials with standardized and clinically meaningful outcome measures.

## Figures and Tables

**Table 1:** Main reasons for exclusion after full-text assessment. The table summarizes the categories of exclusion applied during the full-text screening phase, providing an overview of the most frequent causes for study ineligibility.

Reason for Exclusion	Number of Studies
Narrative or systematic review	18
Not within scope of the re-view	27
Studies not relevant to the research question	1
No abstract available/insufficient data	30
Not adherent to inclusion criteria	2
Total	78

**Table 2:** Characteristics of the sources of evidence included in this scoping review. The table reports study design, clinical indication, type of grafting material, crystalline phase, comparator, follow-up duration, and main clinical, radiographic, and histological outcomes, highlighting the heterogeneity of indications and methodologies across the included trials.

Author, Year of Publication	Study Design	Graft Materials Evaluated	Description	Number of Patients	Time of Follow-Up	Key Findings	Complications Reported	Results	Specific Material Phase
Pepelassi et al., 1991 [24]	RCT	Tricalcium phosphate (TCP; phase not specified) + plaster of Paris + doxycycline composite graft.	The study compared a composite graft (TCP + plaster of Paris + doxycycline) with debridement alone in periodontal furcation defects. In 15 patients, grafted sites showed greater bone fill and clinical improvement after 6 months, especially in class III defects.	15	6 months	Composite graft yielded superior defect fill and clinical outcomes compared with debridement.	None reported; the absence of clinical inflammation and infection during healing confirmed biocompatibility.	The composite graft has potential in promoting healing of furcation lesions, with plaster of Paris serving as a binder and doxycycline carrier.	Tricalcium phosphate (TCP; α/β phase not specified)
Nery et al., 1990 [25]	RCT	Biphasic calcium phosphate (BCP; HA/TCP ratio not specified; TCP phase not specified)	Randomized clinical trial in 137 patients comparing biphasic calcium phosphate (BCP), autogenous graft, and open-flap curettage for periodontal defects. After 3-year follow-up, no significant differences were found; all treatments showed similarly limited clinical improvement.	137	3 years	The biphasic calcium phosphate (BCP) ceramic group showed slightly greater clinical attachment gain (1.0 mm) vs. OFC (0.9 mm) and autogenous bone (0.4 mm); not statistically significant.	Not explicitly reported; plaque and gingival indices increased with time but without intergroup differences.	All three treatments (BCP, autogenous bone graft, and open-flap curettage) were similarly ineffective in improving periodontal outcomes in this veteran population.	Biphasic calcium phosphate (HA/TCP; ratio and TCP phase not specified)
Deshoju et al., 2017 [26]	RCT	Zinc-substituted nanostructured monetite scaffold (Sil-Oss^®^) vs. hydroxyapatite (HA).	The study compared a zinc-substituted monetite scaffold (Sil-Oss^®^) with hydroxyapatite in periodontal intraosseous defects using a split-mouth design. Sil-Oss^®^ showed greater bone fill, while clinical outcomes were similar between groups.	30	9 months (evaluations at 3, 6, 9 months; histology at 7 months)	Sil-Oss^®^ showed greater bone fill and higher mineralized tissue than hydroxyapatite; no differences in CAL or PPD.	No relevant complications reported	Sil-Oss^®^ has potential as a grafting material for periodontal regeneration	Monetite (zinc-substituted; DCPA)
Flores Fraile et al., 2020 [27]	RCT	Synthetic composite graft: monetite + silica gel + PS-wollastonite + calcium-deficient hydroxyapatite vs. Bio-Oss^®^ (bovine hydroxyapatite).	RCT on 33 patients assessing a novel synthetic graft for post-extraction socket preservation versus Bio-Oss^®^. The new material was safe and showed higher resorption and improved bone regeneration.	33	180 ± 5 days (~6 months), with check-ups at days 9, 21, 42, 84, and 175.	Both Sil-Oss^®^ and Bio-Oss^®^ preserved alveolar ridge dimensions; Sil-Oss^®^ showed lower radiodensity but greater new bone formation and osteoid activity than Bio-Oss^®^.	Pain and inflammation were reported by the majority of patients at the first follow-up (about 85% and 70% respectively), but these were attributed to surgical trauma and resolved without further complications.	Both materials can be safely used and were equally effective in maintaining alveolar dimensions. They were shown to be biocompatible, bioactive, and osteoconductive. The experimental material had the additional advantage of being resorbable and replaced by new bone, enhancing bone regeneration.	Composite including monetite (DCPA) + calcium-deficient HA
Kesmas et al., 2010 [28]	Prospective cohort study	Biphasic calcium phosphate (BCP; HA/TCP ratio not specified; TCP phase not specified) + resorbable collagen membrane.	Evaluated ridge preservation in maxillary incisor sites using biphasic calcium phosphate and a collagen membrane. Minimal ridge reduction and variable new bone formation were observed after 4 months, with few residual particles.	8	4 months until implant placement, with follow-up extending up to 12 months post-implant.	BCP with a resorbable collagen membrane preserved alveolar ridge contour over 4 months, supported new bone formation, and allowed implant placement. Some graft remnants persisted, but gradual resorption and remodeling occurred, reducing post-extraction ridge loss.	Healing was uneventful in all cases, with no infections or significant inflammatory complications reported.	Ridge preservation with BCP and a collagen membrane appears to be a safe and effective alternative for maintaining alveolar ridge dimensions prior to implant placement, offering favorable clinical, radiographic, and histological outcomes.	Biphasic calcium phosphate (HA/TCP; ratio and TCP phase not specified)
Tamimi et al., 2010 [29]	Prospective split-mouth controlled clinical pilot study	Monetite granules vs. bovine hydroxyapatite (BH).	Monetite and bovine hydroxyapatite were compared in post-extraction sockets using a split-mouth design. Monetite showed higher new bone formation and lower residual graft after 6 months.	5	6 months (biopsy at implant placement; additional implant follow-up 3–6 months)	Monetite produced significantly more new bone (59.5%) compared to bovine hydroxyapatite (33.1%); less residual graft material remained with monetite	No major adverse events; normal healing observed	Monetite is more resorbable and supports better bone regeneration compared to bovine hydroxyapatite	Monetite (dicalcium phosphate anhydrous; DCPA)
Baldock et al., 1985 [30]	Case series	Tricalcium phosphate (TCP; phase not specified)	TCP was implanted in periodontal defects; clinical improvements were observed, but histology showed connective-tissue encapsulation of particles with minimal new bone or cementum formation.	2	3, 6, and 9 months (block biopsies at these times)	TCP implants were well tolerated and encapsulated by connective tissue; limited new bone growth; some new cementum observed but minimal new attachment	No abnormal postoperative sequelae; minor soft tissue exposure in some sites but healed normally	TCP was biocompatible and safe but did not stimulate significant new bone or attachment; limited regenerative potential	Tricalcium phosphate (TCP; α/β phase not specified)

## Data Availability

Data are available upon request due to privacy.
